# GENTLE: a novel bioinformatics tool for generating features and building classifiers from T cell repertoire cancer data

**DOI:** 10.1186/s12859-023-05155-w

**Published:** 2023-01-30

**Authors:** Dhiego Souto Andrade, Patrick Terrematte, César Rennó-Costa, Alona Zilberberg, Sol Efroni

**Affiliations:** 1 Bioinformatics Multidisciplinary Environment (BioME), Metropole Digital Institute (IMD), Federal University of Rio Grande Do Norte (UFRN), Natal, 59078-970 Brazil; 2 The Mina & Everard Goodman Faculty of Life Sciences, Bar-Ilan University, Ramat-Gan, Israel

**Keywords:** T cell receptor repertoire, Feature selection, Machine learning tools

## Abstract

**Background:**

In the global effort to discover biomarkers for cancer prognosis, prediction tools have become essential resources. TCR (T cell receptor) repertoires contain important features that differentiate healthy controls from cancer patients or differentiate outcomes for patients being treated with different drugs. Considering, tools that can easily and quickly generate and identify important features out of TCR repertoire data and build accurate classifiers to predict future outcomes are essential.

**Results:**

This paper introduces GENTLE (GENerator of T cell receptor repertoire features for machine LEarning): an open-source, user-friendly web-application tool that allows TCR repertoire researchers to discover important features; to create classifier models and evaluate them with metrics; and to quickly generate visualizations for data interpretations. We performed a case study with repertoires of TRegs (regulatory T cells) and TConvs (conventional T cells) from healthy controls versus patients with breast cancer. We showed that diversity features were able to distinguish between the groups. Moreover, the classifiers built with these features could correctly classify samples (‘Healthy’ or ‘Breast Cancer’)from the TRegs repertoire when trained with the TConvs repertoire, and from the TConvs repertoire when trained with the TRegs repertoire.

**Conclusion:**

The paper walks through installing and using GENTLE and presents a case study and results to demonstrate the application’s utility. GENTLE is geared towards any researcher working with TCR repertoire data and aims to discover predictive features from these data and build accurate classifiers. GENTLE is available on https://github.com/dhiego22/gentle and https://share.streamlit.io/dhiego22/gentle/main/gentle.py.

**Supplementary Information:**

The online version contains supplementary material available at 10.1186/s12859-023-05155-w.

## Background

Identifying high-quality biomarkers in cancer data is a formidable challenge, but TCR repertoires have shown to be a useful source in surmounting this obstacle [1, 2]. TCRs are generated by a VDJ (variable, diversity, joining) recombination process that can generate a potential diversity of 10^19^ unique TCRs [3]. This process yields two protein chains consisting primarily of alpha and beta chains; in approximately 10 percent of cases, they consist of gamma and delta chains [4]. The beta chain uniquely contains the diversity (D) gene segment, which includes greater diversity–specifically in the CDR3 (Complementarity Determining Region 3) region where the D gene segment is located. Despite its complexity, a sample of these TCR repertoires generates substantial insights into immune system behavior, thus providing important information regarding the choice of therapy [5].

Many features of the repertoire, such as diversity, clonality, motifs, and network presentation, are often used as metrics to distinguish between cancer patients who may respond to a certain drug or simply between sick and healthy individuals [6–8]. Table 1 provides additional information about the terms introduced above. Originally used in ecology, diversity metrics can be adapted to characterize the TCR repertoire. The Shannon and Simpson indices are the most commonly used metrics [9], although other metrics, including Hill numbers, Pielou, and Gini, also serve as strategies [6, 9]. We previously identified [10] significant differences of 1 minus Pielou (referred to as clonality in the article; see their supplemental information file) and Simpson indices between control and mice with mammary tumors after immunotherapy. Network-based approaches may consider each TCR as a node and utilize distance metrics to build edges between the nodes. In another work [11], we demonstrated that a network representation could stratify control and transgenic mice blood samples. A longitudinal analysis of TCR repertoire networks showed variations in the density index of the network. The Levenshtein metric was employed to represent the editing distances used to build the networks. Using physicochemical motifs, Ostmeyer and collaborators [12] distinguished tumor tissue from patient-matched healthy tissue in the same organ by extracting 4-mers from CDR3 sequences and using their frequencies to build high-accuracy classifiers for breast cancer and colorectal cancer. Additionally, Wang and colleagues [13] identified 11 structural motifs to distinguish long-term survivors from short-term survivors with nasopharyngeal carcinoma.

**Table 1:** Summary of main TCR repertoire metrics

Term	Definition	Biological significance	Reference
Diversity	The richness of the repertoire; the number of different receptors in the population	Important to the immune system given its ability to mount protective immune responses	[14]
Clonality	The frequency of each T cell in the repertoire based on its receptor	Serves as evidence–a form of molecular fingerprint–to identify the origin of certain disorders	[15]
Motif	Short sequence of amino acids which may determine the affinity of a TCR to an antigen	Critical for recognition of certain antigens	[16]
Network	A visual representation in which clones are associated with vertices and edges are associated with a distance measure between two clones	It captures the relationships between the clones and offers a visualization of the repertoire’s structure	[11]

Recent technological breakthroughs have advanced the entire field [17]. In tandem with progress in immunotherapy, great improvements in NGS (Next Generation Sequencing) options now allow the sequencing of longer reads in large quantities and at an affordable price [17]. These improvements have contributed to the gradually increasing availability of TCR-based data, while data analysis tools are still limited. TCR repertoire-oriented tools can be used in two types of analysis: low-level or high-level. Low-level tasks involve raw data processing, while high-level tasks extract information from processed data [18]. IMGT/HighV-QUEST [19], IgBLAST [20], MiXCR [21], and MiTCR [22] are some of the most cited tools for low-level tasks, while Table 2 summarizes the most cited tools for high-level tasks and depicts their main features. The experimental design should guide the adequate tool selection according to a specific purpose. GENTLE generates features and allows users to create classifiers and evaluate their predictive power on experimental samples, thus meeting the need for fast and easy-to-use data analysis tools developed using–and based on–easily accessible sources [23].

**Table 2:** Summary of high-level computational tools for TCR analysis

Tools	Input data	Implementation	Open-source	Analysis
GENTLE	AIRR-seq data that are labeled on the repertoire level	Python Streamlit library	Yes	Diversity, network, motif, dimensionality reduction, Normalizations, feature selection, and classifier methods
ImmuneML [24]	AIRR-seq data that are labeled on the repertoire level or sequence level	Python Command line Galaxy web app	Yes	Data simulation, classifiers, and parameter tune
Scirpy [25]	scRNA	Python package	Yes	Diversity, clonotype analysis, spectratype, dimensionality reduction and query epitope
Immunarch [26]	scRNA/bulk	R package	Yes	Diversity estimation, dimensionality reduction, and clustering methods
ImmunoSEQ [27]	Assay to be sequenced	Service web tool	No	Classifiers and data sharing
Immcantation [28, 29]	Various data formats	Python R packages	Yes	Clonal lineage, clonal clustering, repertoire diversity, VDJ gene usage and phylogenetic analysis
VDJtools [30]	Various data formats	Java	Yes	Diversity analysis, repertoire overlap, repertoire clustering, clonality filtering and annotation, and visualization
CoNGA [31]	Various data formats	Python package	Yes	Expression and TCR by a graph-based approach, dimensionality reduction and visualization
scRepertoire [32]	Contig outputs from the 10 × Genomics Cell Ranger	R package	Yes	Clonotypes analysis, visualizing contigs, clonal space homeostasis, proportion, overlap analysis, diversity, clustering, dimensionality reduction, alluvial and chord diagrams
ImmuneRef [33]	AIRR-seq data	R package	Yes	Analysis of repertoire similarity across repertoire features, calculates overlap, analyzes repertoire global and local similarities, and visualizes results with clustered heatmaps for each layer and a multidimensional similarity network

In summary, we have developed GENTLE, the first tool to offer a machine learning pipeline for TCR repertoire cancer data: it enables the generation of features, normalization methods, feature selection algorithms, classifiers construction, and evaluation metrics for internal and external validation.

## Implementation

### Architecture

The code for GENTLE was written in Python 3 and can be run on version 3.9 or higher. We used Streamlit to construct the GUI (Graphical User Interface), Pandas for data manipulation, Plotly for data visualization, and scikit-learn for many machine learning algorithms [34]. Streamlit was chosen for its speed and simplicity in implementing an application front-end and for easily and freely deploying and sharing the application with the community. Streamlit also makes available some functions that do not run unless a specific parameter has changed. In light of this, we incorporated these functions to avoid redundant calculations and to keep the program running fast. The source code of GENTLE is available on https://github.com/dhiego22/gentle; the README documentation is succinct and clear for users, and the program can be easily installed with virtualenv or docker.

### General flow

The input into GENTLE is a.csv file format. For files that surpass the maximum size supported (200 MB), the.csv file can be zipped and uploaded in a zip format. The file must be a dataframe in which the rows represent the samples of the experiment, and the columns represent the TCRs with one additional column representing the label of each sample (e.g., case/control). The values should represent the counts of the TCRs in the samples. We provide examples of the input data in the Github repository.

After uploading the data, four options will appear in the sidebar; these options are the feature dimensions to be analyzed. It is important to emphasize that when uploading a different dataframe, one should always erase the cache from the options menu in the top-right corner of the screen. The **Diversity** metric calculates popular diversity measurements widely used in ecology such as richness, Shannon, Simpson, inverse Simpson, Pielou, one minus Pielou, hill numbers and Gini indices. The **Network metric** will use the TCR sequences as nodes and calculate a Levenshtein distance of two, according to [11], to create edges between the nodes. After creating the networks, features like the number of nodes and edges, density, clustering coefficient, transitivity and connected components are calculated. The **Motif** metric calculates the frequency of contiguous letters specifically, 2-mers, 3-mers and 4-mers. Finally, GENTLE gives the option to use six different **dimensionality reduction** methods: PCA (Principal Component Analysis) [35], t-SNE (t-distributed Stochastic Neighbor Embedding) [36], UMAP (Uniform Manifold Approximation and Projection) [37], ICA (Independent Component Analysis) [38], SVD (Singular Value Decomposition) [39], and ISOMAP (Isometric Mapping) [40]. In addition, when exporting the features as a dataframe, GENTLE offers three normalizing options in the sidebar. The first option is standard normalization, which converts the data to an average value of 0.0 and a standard deviation of 1.0. The second option is min–max normalization, which linearly converts the data such that the minimum value is − 1.0 and the maximum value is 1.0. The third option is robust scaler, which subtracts the median value and linearly scales the data based on the interquartile range. Normalization is optional, but it can significantly impact the algorithmic performance [41].

GENTLE provides four feature selection methods: Pearson’s correlation, Ridge, XGBoost, and mRMR. Upon selecting the methods, a dataframe will be created with a rank of the features with the greatest predictive power, according to each method, where zero means the feature was not selected by the method; one means the feature was selected as the most predictive; two, the second-most predictive; and so on. It is important to emphasize that Pearson's method considers only one feature at a time when defining its predictive power; this way, the two most predictive features can be so correlated that their combination may not improve a classifier's predictive power when trained together. In contrast, the other methods will consider the combination of the features, which means the two most predictive features are the combination of two features that will produce the most predictive classifier if trained together. In addition, for visualization purposes, one can choose two featuresto display a 2D scatter plot, or three featuresto display a rotating 3D scatter plot.

For the next step, one can perform the classification and validate the predictive power of the selected features. Four classifiers can be chosen: GNB (Gaussian Naive Bayes), LDA (Linear Discriminant Analysis), LR (Logistic Regression), and DT (Decision tree). A radar plot will be generated, representing the five main scoring methods for classifiers: accuracy, precision, recall, F1, and AUC (Area Under Curve) ROC (Receiver Operating Characteristics) curve.

Finally, one can upload another dataset for external validation purposes. This ultimate step produces a confusion matrix and a radar plot with accuracy, precision, recall, F1-score and AUC ROC scoring methods as explained above.

**Fig. 1:**
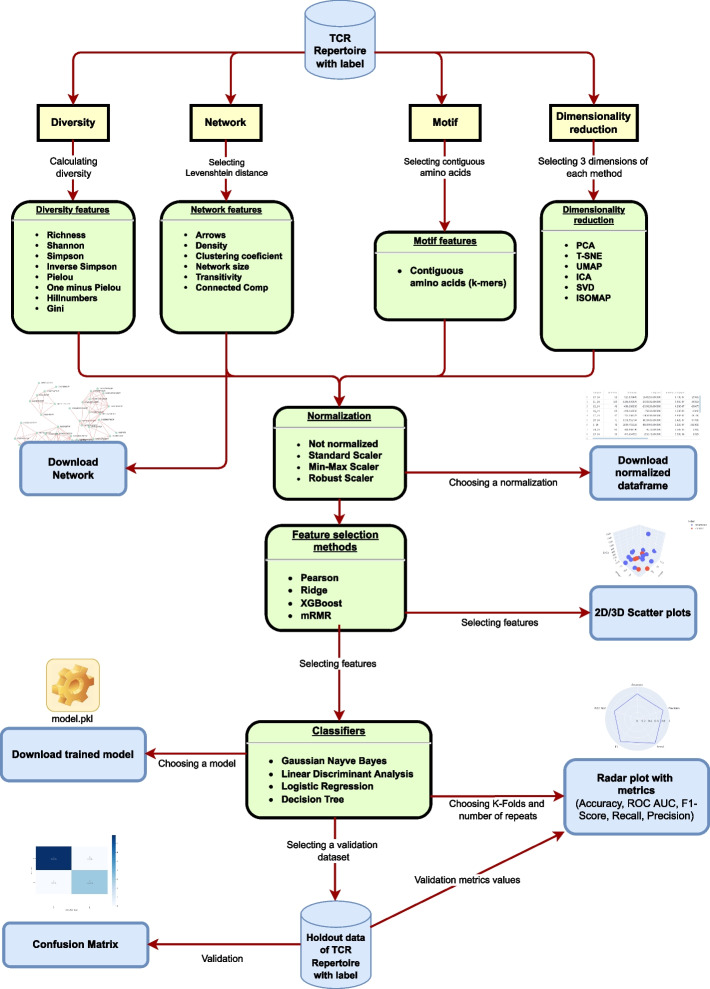
GENTLE Workflow

Each dataframe generated can be downloaded (in.csv file format), along with the networks, the charts (in.png image format), and the classifier model (in pickle format). The networks created can also be visualized. GENTLE can be used for educational purposes due to its user-friendly interface and simplicity. This tool is particularly useful in providing fast feedback when analyzing a TCR repertoire and its features. Figure 1 summarizes the main steps for using GENTLE and understanding its capabilities. There is also a concise walkthrough with screenshots in 11 steps available in the Additional file 1—A Walkthrough of GENTLE and the methods from GENTLE’s flow are summarized in the Additional file 2—Diversity metrics, network metrics, dimensionality reduction, classifiers and scoring metrics.

### Algorithms for feature selection and classification

Pearson’s correlation is a widely applied strategy to find the features most related to a target and to eliminate any redundancies between features [42]. It provides a rank of the most correlated features according to a given target; it does not consider a set of features for prediction but rather considers features independently. For example, if feature A and feature B have the highest correlation values but are alike, we can achieve the same results with only one. mRMR (minimum redundancy maximum relevance) circumvents this issue by selecting features with high predictive power which are simultaneously different from one another; i.e., it selects the smallest relevant subset of features [43]. Ridge regression (also known as L2 regularization) is the ideal method to tackle the overfitting issue, given its regularization use. This method determines variables with zero effect on the data without wasting predictive information. XGBoost (Extreme Gradient Boosting) uses ensembles of decision tree methods, like gradient boosting, to estimate the importance of the features when training a predictive model. It is an appropriate method when working with large datasets; moreover, it can reflect complex interactions between the features [44]. Here, we adopt algorithms belonging to the three main classes of feature selection methods: filter-based represented by Pearson’s correlation, embedded represented by Ridge and XGBoost, and wrapper represented by mRMR.

To build the classifiers, we chose four methods based on their transparency, speed, and capacity to compete with sophisticated methods (e.g., neural networks, ensemble methods) and degree of relevance in the literature. In terms of transparency, we are referring to internal processes used and the various weighted factors that remain unknown, commonly known as a ‘black box’ algorithm. **Naive Bayes** could classify between malignant and benign breast cancer using the Breast Cancer Wisconsin Data Set, providing fast and accurate results [45]. **Linear Discriminant Analysis** is widely used in the biomedical field to separate groups according to disease and response to treatments. It is an efficient approach to dealing with the small sample size problem [46]. **Logistic Regression** was applied to calculate the log-likelihood ratio of radiological response to anti-PD1 (programmed cell death protein 1) therapy TCR repertoire data of patients with metastatic melanoma [47]. **Decision Tree** was the base method used to investigate features in 15 patients with NSCLC (non-small cell lung cancer) using a combination of exome, transcriptome, and TCR repertoire data [48].

In summary, the feature selection methods and the classifier models can tackle diverse issues, perform rapidly, and deliver competitive and accurate results compared to other state-of-the-art approaches.

## Results and discussion

### Data description

To perform a case study workflow, we used a public dataset from the TCRdb that can be accessed from the link http://bioinfo.life.hust.edu.cn/TCRdb/#/download (access the project PRJNA297261). The website contains seven TCR repertoire projects, but only two of them contain at least two conditions: ‘Healthy’ or ‘Breast Cancer’. From these two projects, the immunoSEQ20 project is unbalanced, as it contains 60 ‘Breast Cancer’ samples and only three ‘Healthy’ samples. Considering that, we decided to move forward with the PRJNA297261 project, which was balanced. Notice that the dataset here is only used for demonstration purposes and not for its medical/biological merit. We did not produce this dataset. This database provides preprocessed dataframes that require less processing to fit GENTLE’s input. The original project labels each sample with the condition of ‘Breast Cancer’ or ‘Healthy’ tissues; the project also labels each sample with the cell type of ‘TRegs’ or ‘TConvs’. We made available on Github the script in which we processed the data extracted from the website, turning it into a dataframe that serves as input for GENTLE. This script splits the original project (PRJNA297261) into two dataframes; one with only the TRegs samples, and the other one with only the TConvs samples. Each dataframe labels its samples as ‘Healthy’ or ‘Breast Cancer’. The processed data is summarized in Table 3 and is available on the Github page. The original data comprises TCR beta chain repertoire of regulatory and conventional T cells in peripheral blood from breast cancer patients and healthy individuals. TRegs are important for the regulation of immune response, including TConvs, which can differentiate into effector cells and respond to non-self antigens. Although TRegs and TConvs have different functions they can descend from common clones [49].

**Table 3:** Description of datasets on TCRs available

Filename	Number of different TCRs	Number of samples	Healthy individuals	Breast cancer patients
TRegs.csv	3387	11	3	8
TConvs.csv	23,779	6	3	3

### Case study

In this case study, we identified the features that can separate the healthy from the sick samples in both datasets, and used them to build classifiers. To avoid overfitting, we limited the number of features according to the minimum number of samples found in each category minus one [50]. When building the classifiers, we considered two features: one less from the three healthy patients from the TRegs and in both categories from the TConvs. We trained one model with the TRegs dataset using the TConvs dataset as the test/unseen data; we also made a switch by training one model with the TConvs dataset and using the TRegs dataset as the holdout/unseen data, thus allowing us to analyze the predictive power of each feature for the TCR repertoire.

**Fig. 2:**
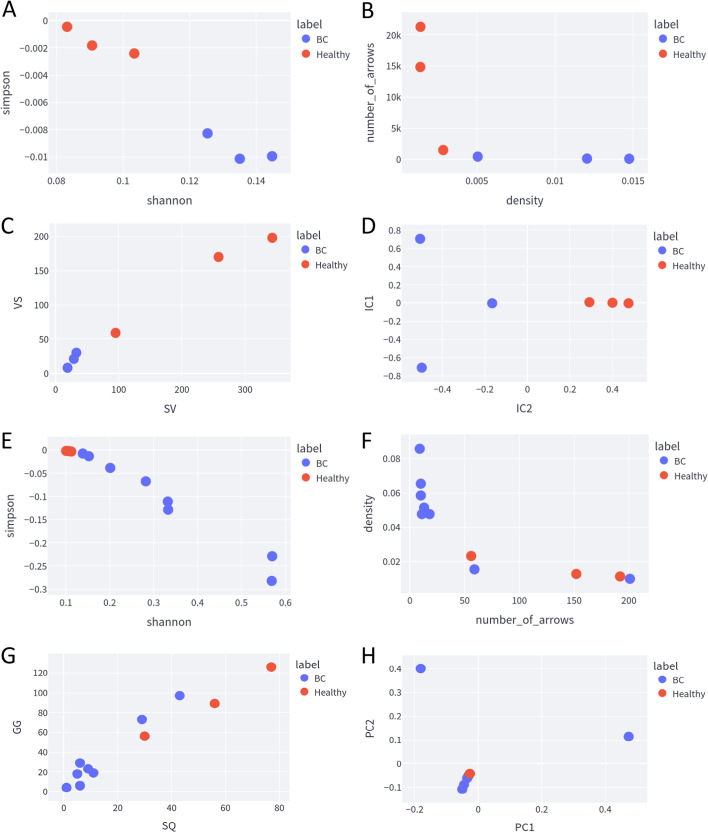
**A**–**D** Scatter Plot of each dimension using the TConvs dataset. **E**–**H** Scatter Plot of each fdimension using the TRegs dataset. It is important to emphasize that we used the most predictive features on each scatter plot, according to the feature selection methods

By analyzing all four dimensions (diversity, network, motif, and dimensionality reduction) using the TConvs dataset, we could construct scatter plots that portray a separation between the healthy and the sick samples. The features in the *x* and *y*-axis of each scatter plot were chosen based on the feature selection method’s choice for the most predictive features. Both Simpson and Shannon indices from the diversity features were able to separate the healthy and sick samples (Fig. 2A), wherein the sick samples had higher values for the Shannon index and lower values for the Simpson index; the opposite was true for the healthy samples. The density and the number of arrows could also accurately separate the samples (Fig. 2B), in which the healthy samples had lower density values and more arrows from the built networks than the samples with breast cancer. The frequencies of the motifs ‘VS’ (valine followed by a serine) and ‘SV’ (serine followed by a valine) could likewise separate the samples (Fig. 2C); both frequencies were more common in healthy patients than in sick patients. Many dimensional reduction methods were also able to separate the samples, but we depicted an interesting scenario in which the IC1 feature did not, but the IC2 feature completely separated the samples (Fig. 2D). The features generated from these repertoires clearly distinguished the sick and healthy samples as seen in the scatter plots in Fig. 2A–D.

The TRegs dataset could not distinguish the healthy from the sick samples as the TConvs did. The only feature that perfectly separated the samples was the Shannon index (Fig. 2E). Again, density and number of arrows proved to be the most predictive features from the built networks (Fig. 2F). Although they did not completely separate all the samples, they showed a slight tendency towards separation. The combination of the motifs ‘GG’ (two guanines together) and ‘SQ’ (serine followed by a glutamine) shown in (Fig. 2G), can be considered to separate the samples. The dimension reduction methods had difficulty accomplishing this task; PC1 and PC2 are depicted in (Fig. 2H).

It is important to emphasize that the feature selection algorithms chose the features shown in each scatter plot as the ones with the highest predictive power. Many features from the diversity dimension (omitted here) could distinguish the ‘Healthy’ and ‘Breast Cancer’ samples from the TConvs dataset, while even the most predictive features from the network, motif, and dimension reduction had difficulty distinguishing the samples from the TRegs dataset.

**Fig. 3:**
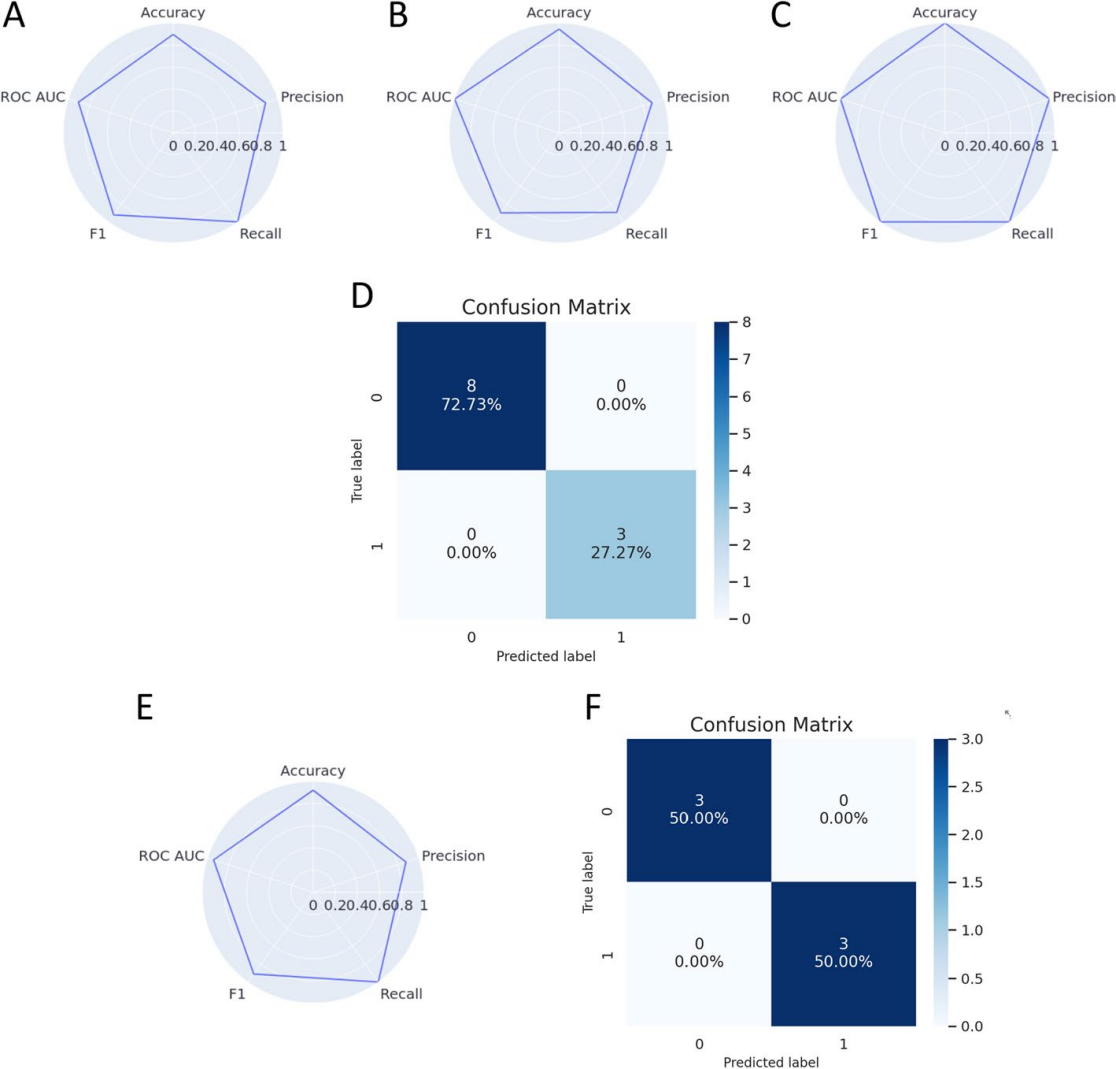
**A**–**C** Stratified validation using threefold and 100 repeats of the classifiers trained with the TConvs dataset where **A** is the Gaussian Naive Bayes, **B** is the Linear Discriminant Analysis and **C** are Linear Regression and Decision Tree classifiers. **D** Confusion matrix of the model trained with the TConvs dataset (train) and validated with the TRegs dataset (test). **E** Stratified validation using threefold and 100 repeats of the Decision Tree classifier trained with the TRegs dataset. **F** Confusion matrix of the model trained with the TRegs dataset (train) and validated with the TConvs dataset (test)

Based on the exploratory analysis we performed (shown in Fig. 2), we decided to build and evaluate the classifiers only with the selected features by the feature selection methods. The Shannon and Simpson indexes were the only features able to build classifiers with high scores of the internal validation and which could classify all the samples correctly from the holdout dataset (see Fig. 3). For the TConvs dataset, all the built models had scores close to 1 with the internal validation, and all of them could predict the samples from the TRegs dataset perfectly. The only model trained with the TRegs dataset that could classify the TConvs dataset perfectly was the Decision Tree.

In sum, this analysis demonstrates the similarity between TRegs and TConvs when analyzing their diversity features, and it portrays how these features can be predictive when analyzing TCR repertoires of breast cancer. Many studies–to cite a few, [2, 51, 52], and [53]–have opted to explore TCR repertoires in their diversity dimension (specifically, the Shannon index). In corroboration with our results, diversity is an essential feature for TCR repertoire analyses and should not be omitted from any relevant discussion. Although this analysis was performed with only a few samples, we believe that it provides an example of the strength of the tool. With the addition of new datasets, more analyses can be done to strengthen the underlying hypothesis regarding information in the repertoire. Both cell types (TRegs and TConvs) are influenced by the same signaling pathways that dictate their development, differentiation, and function [54]. Therefore, understanding TRegs’ and TConvs’ similarities can be a promising pathway to new therapeutic approaches.

## Conclusion

This paper introduces GENTLE, a platform designed to help researchers easily and swiftly analyze their TCR repertoire data. GENTLE provides visualization capabilities and a user-friendly interface.It serves as the first graphical web tool to to incorporate feature selection methods to identify important features built within the TCR repertoire. It also makes available a set of diverse machine learning methods to generate models for classification purposes. The platform makes it possible to compare the performance of the classifiers through the main evaluation metrics for binary classification and also offers metrics for external validation. All data generated by GENTLE can be downloaded for further analysis. As an open-source web application, GENTLE provides researchers tools to analyze data efficiently and to be able to extract biomarkers and build classifiers that could positively improve treatment prospects across healthcare. Our case study showed that diversity features, such as the Shannon and Simpson indices, can be important biomarkers for healthy and sick patients with breast cancer when analyzing their repertoires of TRegs and Tconvs. For future works, we will add features generated from time series data, as some insights can only be gleaned upon analyzing changes in the repertoires over time. Moreover, we will add more options for classifiers and metrics of feature selection aligned more closely with time series data.

## Availability and requirements

Project name: GENTLE. Project home page: https://github.com/dhiego22/gentle & https://share.streamlit.io/dhiego22/gentle/main/gentle.py. Operating system(s): Linux, Windows, Mac. Programming language: Python 3.9 + . Other requirements: Streamlit, Plotly, Pandas, Sckit-learn. License: MIT License. Any restrictions to use by non-academics: None.

## Supplementary Information

**Additional file 1**. A Walkthrough of GENTLE.

**Additional file 2**. Diversity metrics, network metrics, dimensionality reduction, classifiers and scoring metrics.

## Data Availability

The data used in this work was extracted from the TCRdb, a database that contains a plethora of TCR repertoires of many cancer and cell types. The data can be accessed through the link http://bioinfo.life.hust.edu.cn/TCRdb/#/download (project PRJNA297261).

## Abbreviations

GENTLE: GENerator of T cell receptor repertoire features for machine LEarning
TCR: T cell receptor
VDJ: Variable, diversity, joining genes
GUI: Graphical user interface
TRegs: Regulatory T cells
TConvs: Conventional T cells
NGS: Next generation sequencing
CDR3: Complementarity determining region 3
PCA: Principal component analysis
t-SNE: T-distributed stochastic neighbor embedding
UMAP: Uniform Manifold approximation and projection
ICA: Independent component analysis
SVD: Singular value decomposition
ISOMAP: Isometric mapping
GNB: Gaussian Naive Bayes
LDA: Linear discriminant analysis
LR: Logistic regression
DT: Decision tree
AUC: Area under curve
ROC: Receiver operating characteristics
NSCLC: Non-small cell lung cancer
V: Valine
S: Serine
G: Guanine
Q: Glutamine
XGBoost: Extreme gradient boosting
mRMR: Minimum redundancy maximum relevance
PD1: Programmed cell death protein 1
