# Supplementary material for: GENTLE: a novel bioinformatics tool for generating features and building classifiers from T cell repertoire cancer data
Source: BMC Bioinformatics. 2023 Jan 30;24:32. doi: 10.1186/s12859-023-05155-w (PMC9885559; doi:10.1186/s12859-023-05155-w)

A Walkthrough of GENTLE

In this walkthrough, we provide 10 thoroughly explained steps of how to use GENTLE. We show all of the possible analyses and visualizations that the platform allows. The dataset used for this example contains processed TCR repertoires of breast cancer and it is available to be downloaded at http://bioinfo.life.hust.edu.cn/TCRdb/#/download/PRJNA297261.

For this walkthrough, we will be using the data made available on the website (https://github.com/dhiego22/gentle). The data_preprocess.py script uses a comma to save the file, but can be altered according to the user's preferences.

Step 1:

Begin by selecting the type of delimiter used in the file, provided in the multiple choice list. For this example, we will use a comma.

Step 2:

Click on "Browse Files" and select the data frame you would like to upload.

GENTLE: GENerator of T cell receptor repertoire features for machine LEarning

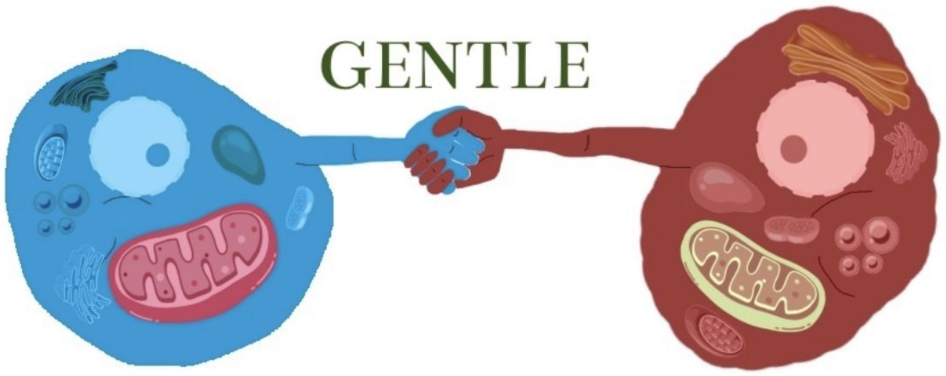

The source code and a quick guide can be found on https://github.com/dhiego22/gentle

1

Upload dataset

Specify the delimiter used in the file

- (selected) ,
- ;
- tab
- space

The dataframe to be uploaded must have the rows as the samples (TCR repertoire) and the columns as the TCR sequences (amino acids) plus the target column. Please set the name of the target column as 'label'. In case your csv file exceed the 200MB size, you can load it as zip.

Drag and drop file here
Limit 200MB per file

2

Browse files

Step 3:

Once you have uploaded your file, a list of options will appear in a sidebar on the left displaying features you can choose to analyze.

A dataframe will appear on the right with the features you have chosen to include.

3

TCR Features

Choose the feature that you want to analyse

- (●) Diversity
- ( ) Network
- ( ) Motif
- ( ) Dimensionality Reduction

Choose the normalization method

No Normalization

Perform feature selection

☐ Check the box to start feature selection process

Diversity Features

Calculating diversity features

Dataframe with diversity features

	sample	richness	shannon	simpson	inverse_simpson	pielou	one_minus_pielou	hillnumt
1	SRR2549130	87	0.333042	-0.128471	7.78E+00	-0.028767	1.028767	7.78E
2	SRR2549132	74	0.332148	-0.110884	9.02E+00	-0.025763	1.025763	9.02E
3	SRR2549133	133	0.282146	-0.067296	1.49E+01	-0.013761	1.013761	1.49E
4	SRR2549128	409	0.112402	-0.002965	3.37E+02	-0.000493	1.000493	3.37E
5	SRR2549147	42	0.569917	-0.228805	4.37E+00	-0.061216	1.061216	4.37E
6	SRR2549144	769	0.152477	-0.013089	7.64E+01	-0.001970	1.001970	7.64E
7	SRR2549145	45	0.568904	-0.282016	3.55E+00	-0.074085	1.074085	3.55E
8	SRR2549136	130	0.201132	-0.038356	2.61E+01	-0.007880	1.007880	2.61E
9	SRR2549143	752	0.100673	-0.001751	5.71E+02	-0.000264	1.000264	5.71E
10	SRR2549141	594	0.106576	-0.001986	5.04E+02	-0.000311	1.000311	5.04E

Uploaded dataframe has `10` columns (features) and `11` rows (samples)

Press the button to download dataframe with diversity features

Features created! Time elapsed (hh:mm:ss.ms) 0:00:00.309935

Dataframe normalized with No Normalization was created

Uploaded dataframe has `9` columns (features) and `11` rows (samples)

☐ Check the box to visualize scaled dataFrame. Warning: depending on the size it can load very slowly

Time elapsed (hh:mm:ss.ms) 0:00:00.004545

Step 4: (optional)

You now have the option to normalize the features.

You can choose your desired normalization method, and to see the data frame, you must check the box indicated that you want a visualization.

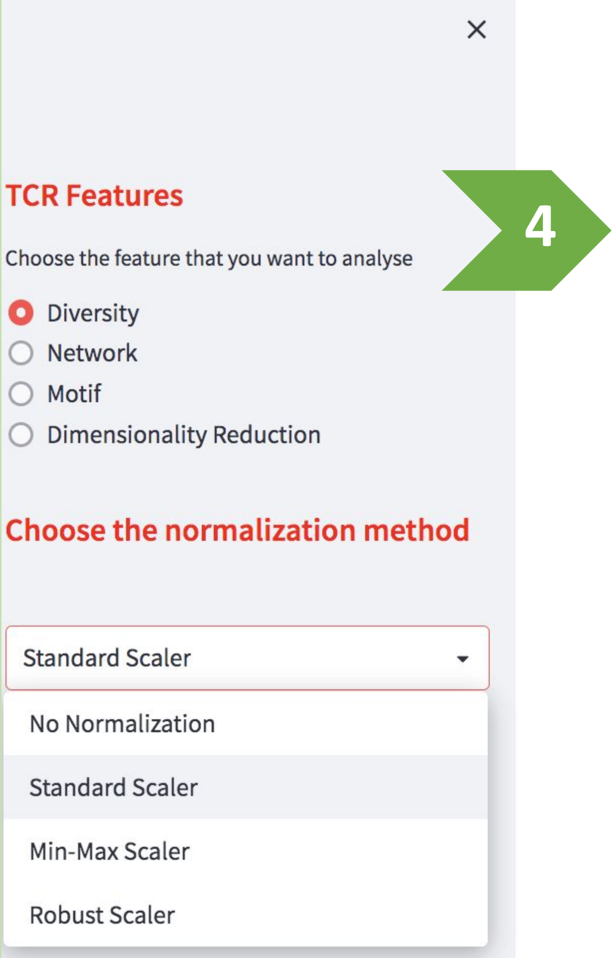

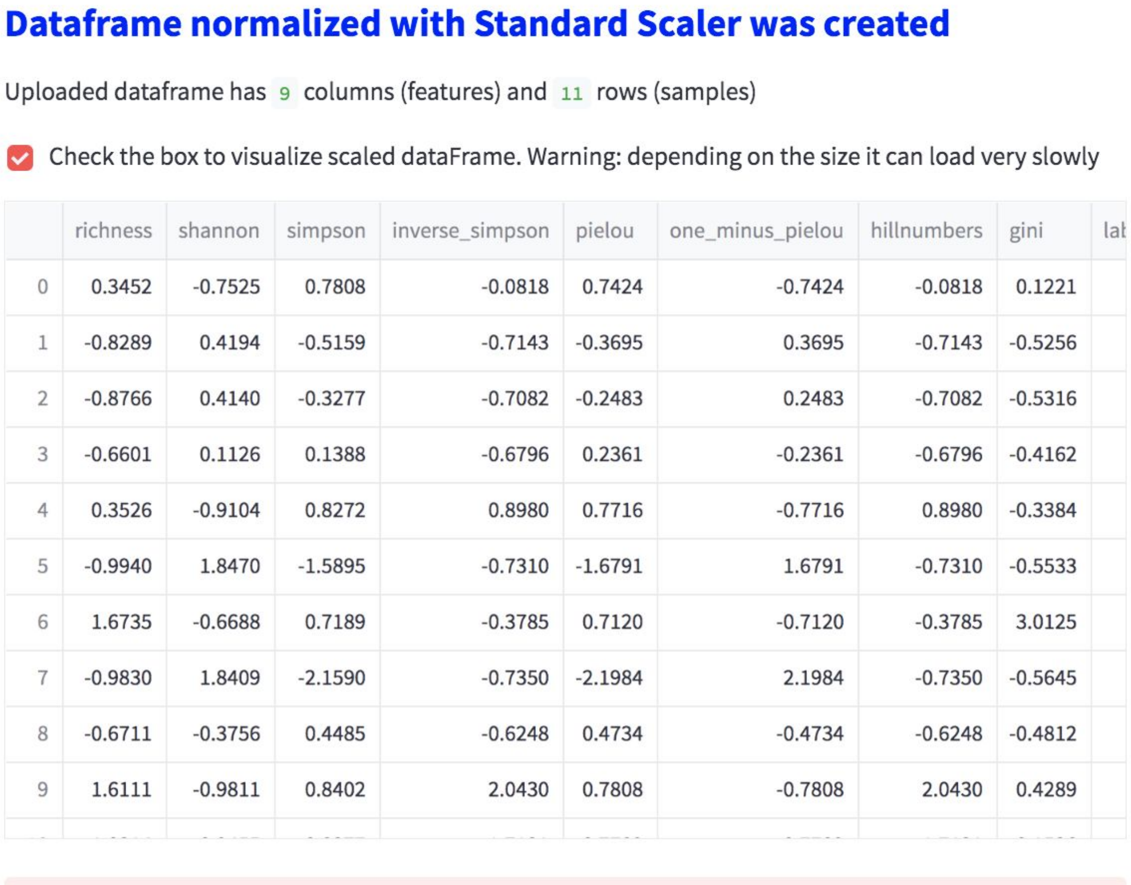

	richness	shannon	simpson	inverse_simpson	pielou	one_minus_pielou	hillnumbers	gini	[illegible]
0	0.3452	-0.7525	0.7808	-0.0818	0.7424	-0.7424	-0.0818	0.1221	
1	-0.8289	0.4194	-0.5159	-0.7143	-0.3695	0.3695	-0.7143	-0.5256	
2	-0.8766	0.4140	-0.3277	-0.7082	-0.2483	0.2483	-0.7082	-0.5316	
3	-0.6601	0.1126	0.1388	-0.6796	0.2361	-0.2361	-0.6796	-0.4162	
4	0.3526	-0.9104	0.8272	0.8980	0.7716	-0.7716	0.8980	-0.3384	
5	-0.9940	1.8470	-1.5895	-0.7310	-1.6791	1.6791	-0.7310	-0.5533	
6	1.6735	-0.6688	0.7189	-0.3785	0.7120	-0.7120	-0.3785	3.0125	
7	-0.9830	1.8409	-2.1590	-0.7350	-2.1984	2.1984	-0.7350	-0.5645	
8	-0.6711	-0.3756	0.4485	-0.6248	0.4734	-0.4734	-0.6248	-0.4812	
9	1.6111	-0.9811	0.8402	2.0430	0.7808	-0.7808	2.0430	0.4289	
[illegible]	[illegible]	[illegible]	[illegible]	[illegible]	[illegible]	[illegible]	[illegible]	[illegible]	

Step 5:

In this step, you must check the box to allow for the feature selection process to take place.

In this example, you can see the results generated on the right.

"0" means that the feature was not selected by the method; "1" means that it was the most predictive feature; "2" means that it was the second-most predictive feature; and so forth.

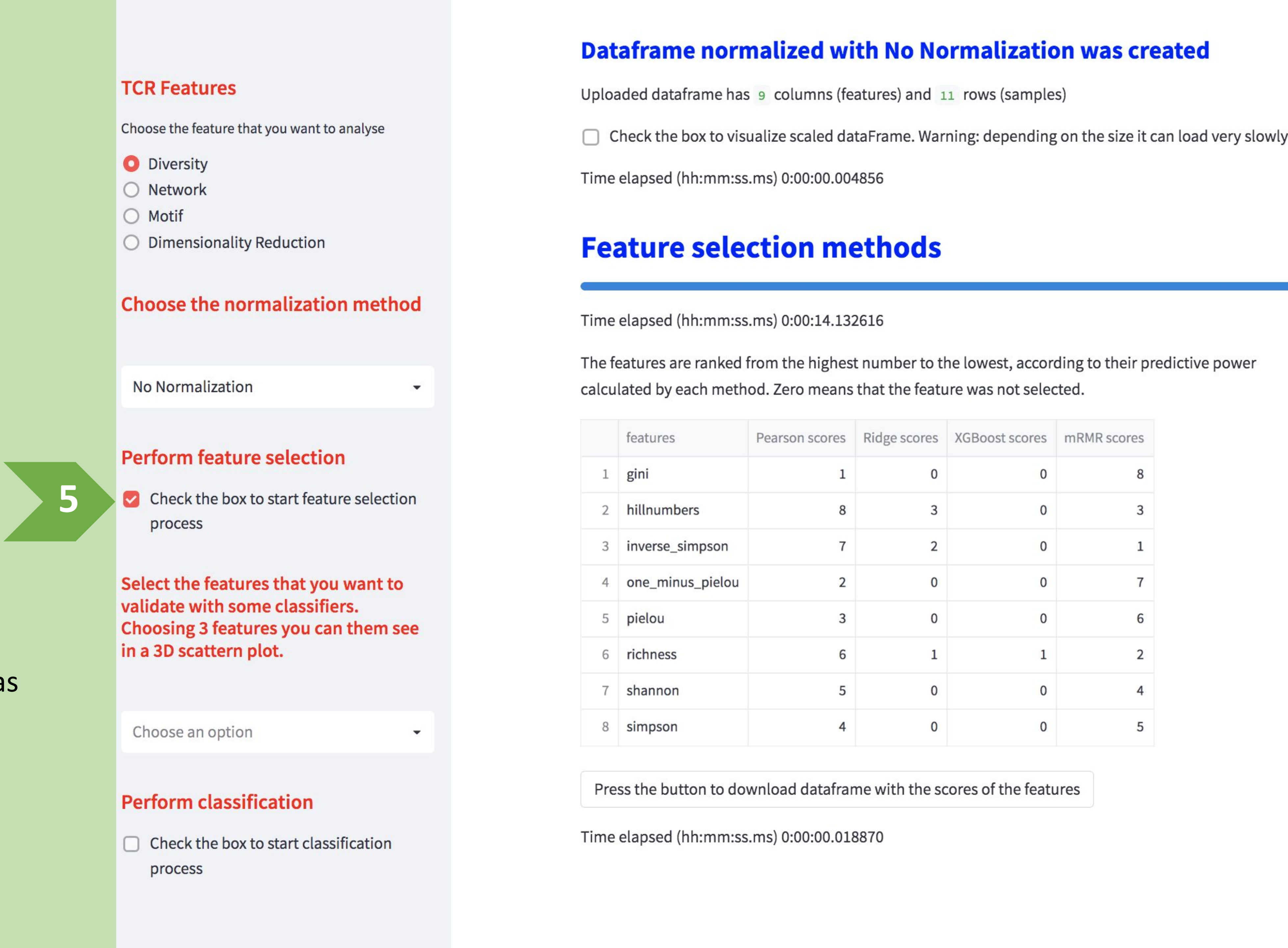

	features	Pearson scores	Ridge scores	XGBoost scores	mRMR scores
1	gini	1	0	0	8
2	hillnumbers	8	3	0	3
3	inverse_simpson	7	2	0	1
4	one_minus_pielou	2	0	0	7
5	pielou	3	0	0	6
6	richness	6	1	1	2
7	shannon	5	0	0	4
8	simpson	4	0	0	5

Step 6:

At this stage, you must select the features which you will use to build your classifiers.

For visualization purposes, if you select two features, you will see a 2D scatter plot appear below the data frame; if you select three or more, you will see a 3D rotating scatter plot.

As you can see, in this example we chose the shannon and simpson indexes.

6

TCR Features

Choose the feature that you want to analyse

- (selected) Diversity
- Network
- Motif
- Dimensionality Reduction

Choose the normalization method

No Normalization

Perform feature selection

- [x] Check the box to start feature selection process

Select the features that you want to validate with some classifiers. Chooseing 2 features you can see a 2D scatter plot. Choosing 3 features you can them see in a 3D rotating scatter plot.

shannon × simpson ×

Perform classification

- [ ] Check the box to start classification process

Feature selection methods

Time elapsed (hh:mm:ss.ms) 0:00:14.132616

The features are ranked from the highest number to the lowest, according to their predictive power calculated by each method. Zero means that the feature was not selected.

	features	Pearson scores	Ridge scores	XGBoost scores	mRMR scores
1	gini	1	0	0	8
2	hillnumbers	8	3	0	3
3	inverse_simpson	7	2	0	1
4	one_minus_pielou	2	0	0	7
5	pielou	3	0	0	6
6	richness	6	1	1	2
7	shannon	5	0	0	4
8	simpson	4	0	0	5

Press the button to download dataframe with the scores of the features

Time elapsed (hh:mm:ss.ms) 0:00:00.015454

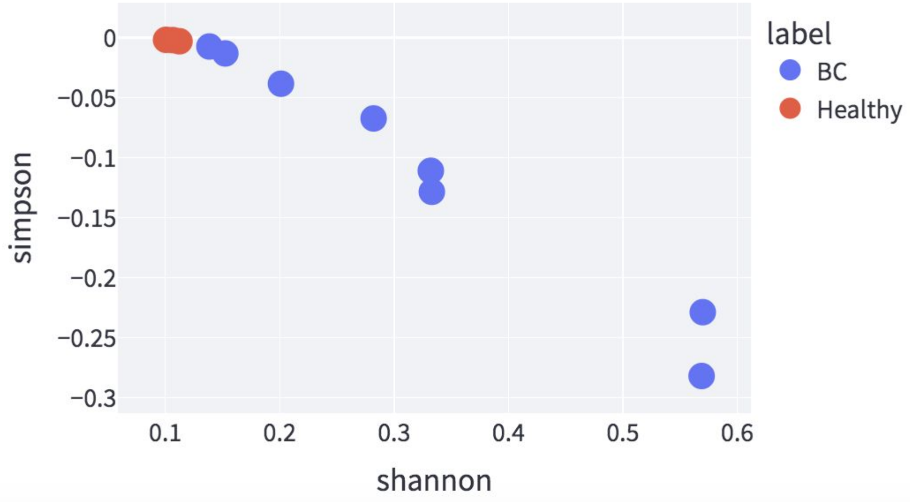

Steps 7-9:

You will now select the number of splits and repeats for the stratified validation. Then select the box “Perform classification” to build a classifer. You can choose one classifier to use amongst the options listed in the sidebar.

After the classifier is built, the five main scoring methods will be calculated and depicted as a radar plot.

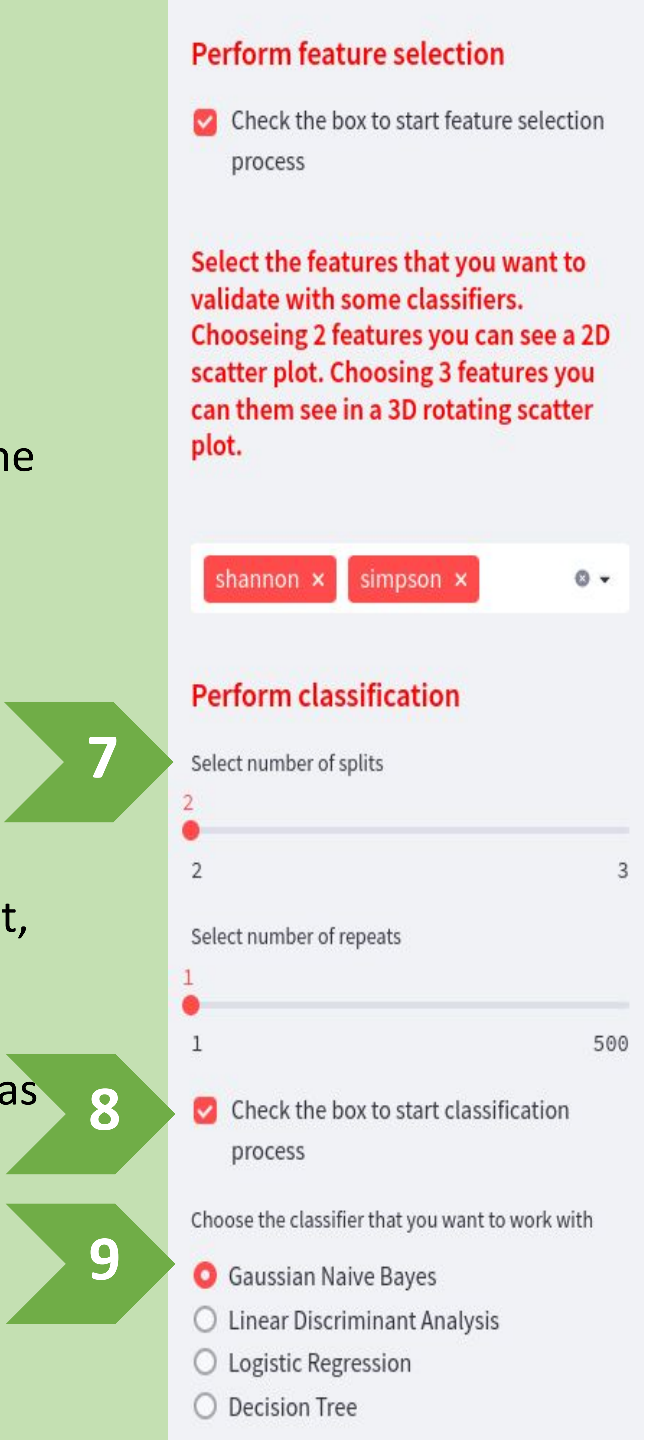

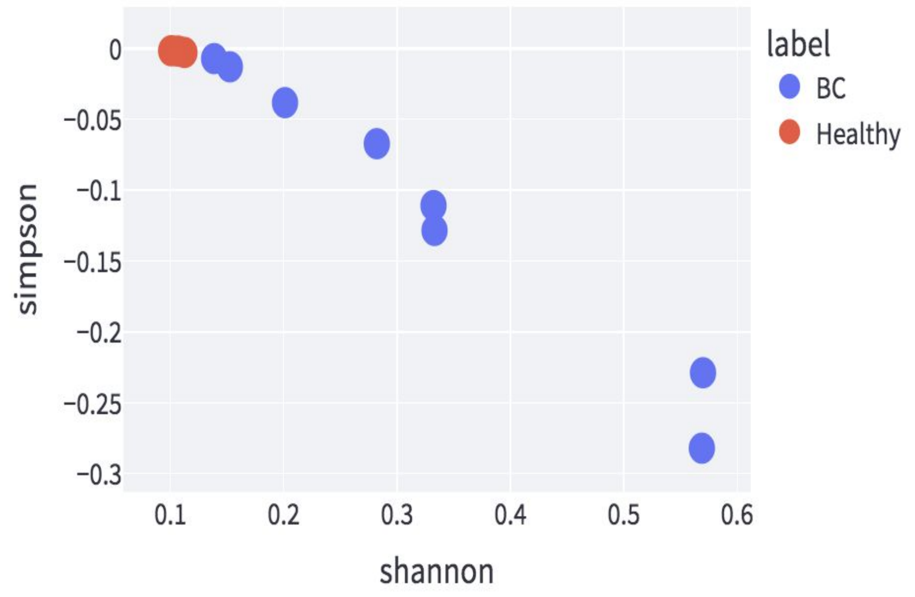

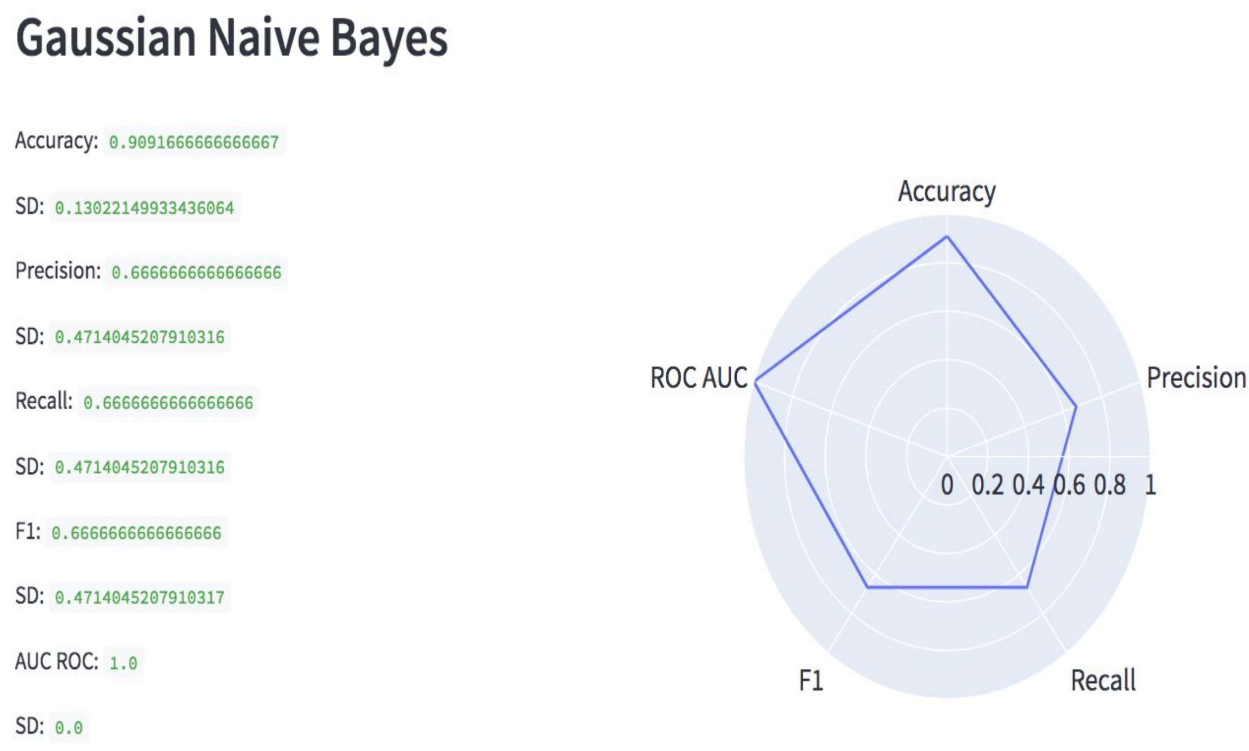

Step 10:

In this step, you will upload a second dataframe for the purpose of validation.

Click on “Browse files” and select the dataframe you wish to upload. The features which you were working with will be calculated automatically, and a dataframe will appear as displayed in the picture.

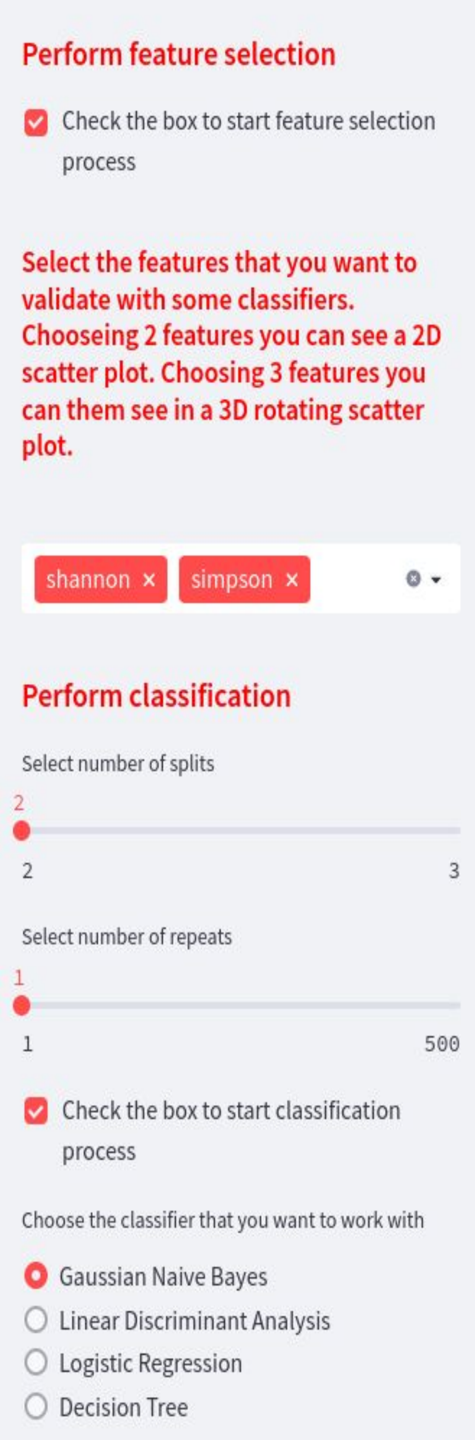

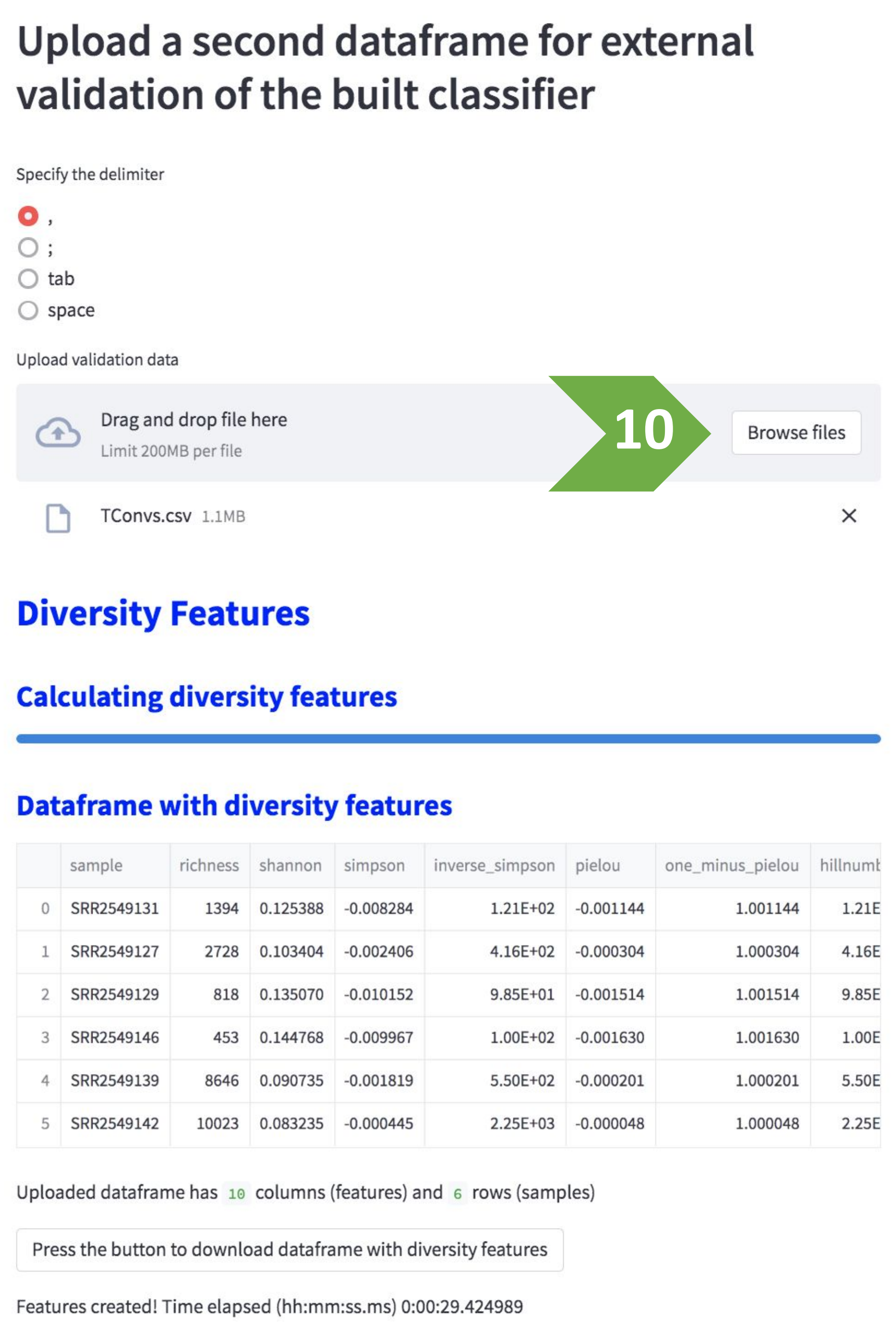

	sample	richness	shannon	simpson	inverse_simpson	pielou	one_minus_pielou	hillnumb
0	SRR2549131	1394	0.125388	-0.008284	1.21E+02	-0.001144	1.001144	1.21E
1	SRR2549127	2728	0.103404	-0.002406	4.16E+02	-0.000304	1.000304	4.16E
2	SRR2549129	818	0.135070	-0.010152	9.85E+01	-0.001514	1.001514	9.85E
3	SRR2549146	453	0.144768	-0.009967	1.00E+02	-0.001630	1.001630	1.00E
4	SRR2549139	8646	0.090735	-0.001819	5.50E+02	-0.000201	1.000201	5.50E
5	SRR2549142	10023	0.083235	-0.000445	2.25E+03	-0.000048	1.000048	2.25E

Step 11:

After uploading the validation dataframe in the previous step, a radar plot with the five main binary classification scoring methods will appear along with a Confusion Matrix.

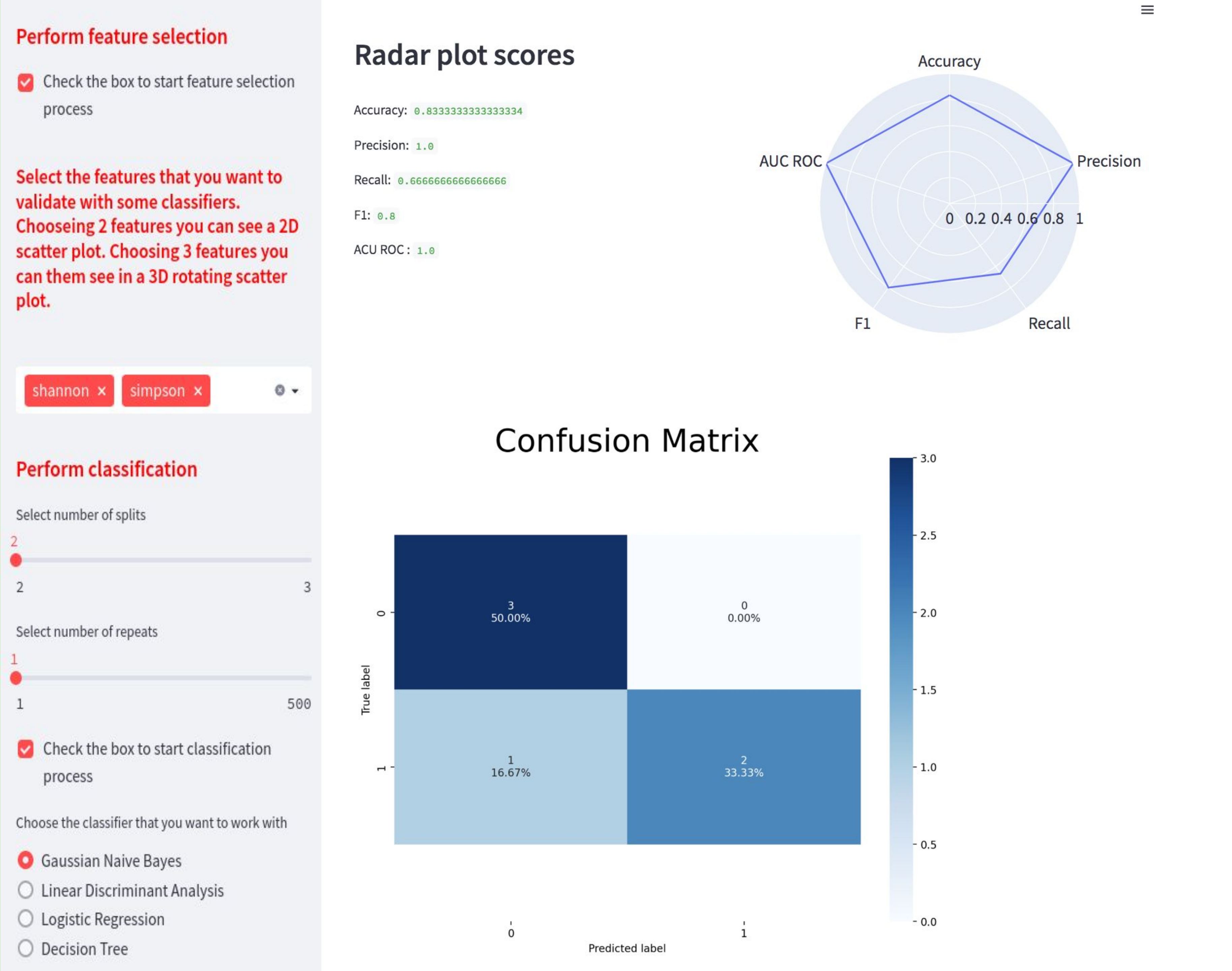

Supplement: Supplementary file 1 — Additional file 1. A Walkthrough of GENTLE. [file 12859_2023_5155_MOESM1_ESM.pdf]
