# Supplementary material for: GENTLE: a novel bioinformatics tool for generating features and building classifiers from T cell repertoire cancer data
Source: BMC Bioinformatics. 2023 Jan 30;24:32. doi: 10.1186/s12859-023-05155-w (PMC9885559; doi:10.1186/s12859-023-05155-w)
Supplement: Supplementary file 2 — Additional file 2. Diversity metrics, network metrics, dimensionality reduction, classifiers and scoring metrics. [file 12859_2023_5155_MOESM2_ESM.pdf]

2 Supplementary File – Diversity metrics, network metrics, dimensionality reduction, classifiers and scoring metrics

2.1 Calculation of repertoire diversity metrics

Richness is a diversity measure of a given set of samples, defined as the total number of unique productive CDR3 amino acid sequences within the TCR β-chain in the dataset of interest.

$$R = \texttt{Total of types or species.} \tag{1}$$

Shannon entropy index quantifies the uncertainty – entropy or degree of surprise – of information [1]. The Shannon of each individual's repertoire is calculated using all sequences of CDR3β:

$$H = -\sum_{i=1}^{R} p_i \; log \; p_i \tag{2}$$

where p_i is probability of each individual clone i, and R is richness.

Simpson index is the proportion of two samples in a set (sampling with replacement) to belong to the same type [2].

$$\lambda = \sum_{i=1}^{R} p_i^2 \tag{3}$$

Inverse Simpson is the effective number of types that is obtained when the weighted arithmetic mean is used to quantify average proportional abundance of types in the dataset of interest. Inverse Simpson's index is used to represent repertoire diversity with high-frequency reads.

$$\texttt{Inv. Simpson} = \frac{1}{\lambda} = \frac{1}{\sum_{i=1}^{R} p(i)^2} \tag{4}$$

Gini indexes measures the inequality among values of a proportion distribution. A Gini coefficient of zero expresses perfect equality, where all values have the same probability. A Gini coefficient of one indicates maximal inequality among types.

$$\texttt{Gini index} = 1 - \lambda = \sum_{i=1}^{R} p_i^2 \tag{5}$$

Pielou's index, or clonal evenness, is the ratio between the Shannon entropy and the maximization of the diversity of individuals type [3]. This measures the level of similarity in the numbers of individuals between different types in a particular environment.

$$\texttt{Pielou's index} = \frac{H_1}{H_{1max}} = \frac{\sum_{i=1}^{R} p_i \; log \; p_i}{log(N)} \tag{6}$$

One minus Pielou is the complement of clonal evenness (1 - Pielou's index).

$$\texttt{1 - Pielou's index} = 1 - \frac{\sum_{i=1}^{R} p_i \; log \; p_i}{log(N)} \tag{7}$$

`Hill numbers`, or effective number of species, is importance of the abundance distribution increases with increasing Hill order [4].

$$ {}^{q}D = \frac{1}{M_{(q-1)}} = \left(\sum_{i=1}^{R} p_i^q \right)^{1/(1-q)} \tag{8} $$

where M_{q-1} is the average proportional abundance of types in the dataset, and p_i is the proportion of type i and q is the Hill order. The Hill number with $q = 0$ is the richness, for $q = 1$, it is the Shannon entropy and for $q = 2$, it is the inverse Simpson index.

2.2 The network metrics

All network metrics are implemented with the python library for Complex Networks [5].

Levenshtein distance, also known as edit distance, is the minimum number of edit (substitutions, insertions, and deletions) necessary to transform one string in another [6]. In TCR context, is number of mutations needed to convert one sequence of amino acids into another.

$$ \texttt{lev}(a,b) = \begin{cases} \mid a \mid & \text{if } \mid b \mid = 0, \\ \mid b \mid & \text{if } \mid a \mid = 0, \\ \texttt{lev}(tail(a), tail(b)) & \text{if } a[0] = b[0], \\ 1 + min \begin{cases} \texttt{lev}(tail(a), b) \\ \texttt{lev}(a, tail(b)) \\ \texttt{lev}(tail(a), tail(b)) \end{cases} & \text{otherwise.} \end{cases} \tag{9} $$

where a and b are sequence of amino acids, $x[n]$ is the n-th amino acid of the sequence x, and $tail(x)$ is the sequence x without the first amino acid.

Density is the ratio between the edges in a graph and the maximum number of edges that the graph can contain.

$$ d = \frac{2m}{n(n-1)} \tag{10} $$

where n is the number of node and m is the number of edges in the graph G.

Clustering coefficient of a node is defined as the probability that two randomly selected nodes are related with each other.

$$ C = \frac{1}{n} \sum_{v \in G} c_v \tag{11} $$

where n is the number of nodes in the graph G.

Transitivity is the ratio of all possible triangles present in a graph G.

$$ T = 3 \frac{\#\texttt{triangles}}{\#\texttt{triads}} \tag{12} $$

where # is the cardinality of a set, and the triads are all possible triangles given by two edges with a shared vertex.

2.3 The Motif metrics

k-mers indicates all the possibles ocurrences of substrings of contiguous amino acids of length k, for k = {2, 3, 4}. A sequence of length L will have $L - k + 1$ k-mers and n^k of total possible k-mers, where n is number of possible monomers amino acids.

2.4 Dimensionality reduction

The methods of dimensionality reduction PCA, ICA, SVD, t-SNE, and t-SNE are implemented using scikit-learn [7]. The UMAP implemented is using umap-learn [8] `https://umap-learn.readthedocs.io`.

Principal component analysis (PCA) is a well known method to reduce the data in a lower dimensional space.

Singular value decomposition (SVD) performs dimensionality reduction in sparse matrices efficiently. Unlike to PCA, it does not not center the data before decomposition. It is also known as latent semantic analysis (LSA).

Independent Component Analysis (ICA) implements FastICA, a fast algorithm based on [9]. The ICA reduce the noise and dimensions by maximizing a measure of non-Gaussianity with statistical independence of the estimated components.

T-distributed Stochastic Neighbor Embedding (TSNE) is a nonlinear dimensionality reduction, in order to model similar objects using nearby points, and dissimilar objects are modeled by distant points [10]. TSNE tries to minimize the Kullback-Leibler divergence between the joint probabilities of the low-dimensional embedding and the high-dimensional data.

Uniform Manifold Approximation and Projection (UMAP) is a dimension reduction technique for general non-linear dimension, and apply a theoretical framework based in Riemannian geometry and algebraic topology [8].

Isometric Mapping (ISOMAP) is a Non-linear dimensionality reduction through Isometric Mapping [11]. The method uses K nearest neighbors to determine the neighbors of each point, incorporates the geodesic distances in a weighted graph, Compute shortest path between two nodes, and calculates lower-dimensional embedding using multidimensional scaling.

2.5 Preprocessing normalizations

MinMaxScaler scales features by scaling each feature to a given range.

$$\mathtt{X}_{scaled}(i) = \frac{\mathtt{X} - min(\mathtt{X}(i)}{max(\mathtt{X}(i)) - min(\mathtt{X}(i))} * (range\_max - range\_min) + range\_min \tag{13}$$

where $\mathtt{X}$ is the matrix of data, i is each column/feature of the data, $range\_min$ and $range\_max$ is the given range of the values.

Standardize scales the mean and scaling to unit variance.

$$z = \frac{\mathtt{X} - \mu}{\sigma} \tag{14}$$

where $\mathtt{X}$ is the matrix of data, μ is the mean of the training samples, and σ is the standard deviation of training samples.

RobustScaler scales feature using statistics that are robust to outliers. It removes the median and scales the data according to the quantile range, in defaults to Interquartile Range (IQR).

2.6 The feature selection

Pearson calculates the correlation for each feature with the label target.

SelectFromModel with Ridge uses a base estimator of Logistic Regression to rank the features based on coefficients weights of features.

SelectFromModel with XGBoost uses a estimator to rank the features based feature importance.

min-Redundancy and Max-Relevance (mRMR) applies mutual information to select features that maximize the statistical dependency on the joint distribution of the target variable [12, 13]. The maximum relevance for the feature set S, given the mutual information of feature f_i in $k-$classes, is:

$$\texttt{maxD}(S,k), D = \frac{1}{|S|}\sum_{f_i \in S} I(f_i, k) \tag{15}$$

The minimum redundancy in the feature subset is given by the sample vectors of pair of features f_i, f_j:

$$\texttt{minR}(S), R = \frac{1}{\mid S^2 \mid}\sum_{f_i, f_j \in S} I(f_i, f_j) \tag{16}$$

This work uses the implementation of Python `https://pypi.org/project/mrmr-selection/`.

2.7 The classifiers

Gaussian Naive Bayes (GNB) performs a probabilistic classification algorithm based on applying Bayes theorem with strong independence assumptions [14].

Linear Discriminant Analysis (LDA) is a classifier linear decision boundary, that apply conditional densities to fit the data using Bayes' rule.

Logistic Regression (LR) implements the traditional well-known classifier with L2 penalty term.

Decision tree (DT) implements the traditional well-known classifier with Gini impurity.

2.8 The scoring metrics of classifiers

The scoring metrics are implemented in Python using scikit-learn [7]. All scores metrics listed here have its best value at 1 and worst score at 0.

Accuracy is defined as the ratio of TCR sequences that are correctly predicted to the positive class.

$$\texttt{accuracy}(y, \hat{y}) = \frac{1}{n_{\text{samples}}}\sum_{i=0}^{n_{\text{samples}}-1} 1(\hat{y}_i = y_i) \tag{17}$$

where $1(x)$ is the characteristic function, y is the vector of target labels, and $\hat{y}$ is the predicted labels.

Precision, or positive predictive value, is the ratio of the positive predictions and the total of positives samples and incorrect predictions of positive class.

$$\texttt{precision} = \frac{\texttt{tp}}{(\texttt{tp + fp})} \tag{18}$$

where `tp` is the number of true positives and `fp` the number of false positives.

Recall, or sensitivity, is the ratio of the positive predictions, and the total of positives samples and incorrect predictions of negative class.

$$\texttt{recall} = \frac{\texttt{tp}}{(\texttt{tp + fn})} \tag{19}$$

where `tp` is the number of true positives and `fn` the number of false negatives samples.

F1 score is a harmonic mean of the precision and recall.

$$\texttt{F1} = 2 * \frac{(\texttt{precision * recall})}{(\texttt{precision + recall})} \tag{20}$$

AUC (Area Under Curve) ROC (Receiver Operating Characteristics) curve is a plot that evaluates the performance of a classification model with two parameters the true positive rate (TPR) – recall – and the false positive rate (FPR).

References

[1] Claude Elwood Shannon. A mathematical theory of communication. *The Bell system technical journal*, 27(3):379–423, 1948.

[2] Edward H Simpson. Measurement of diversity. *nature*, 163(4148):688–688, 1949.

[3] Evelyn C Pielou. The measurement of diversity in different types of biological collections. *Journal of theoretical biology*, 13:131–144, 1966.

[4] Mark O Hill. Diversity and evenness: a unifying notation and its consequences. *Ecology*, 54(2):427–432, 1973.

[5] Aric A. Hagberg, Daniel A. Schult, and Pieter J. Swart. Exploring network structure, dynamics, and function using networkx. In Gaël Varoquaux, Travis Vaught, and Jarrod Millman, editors, *Proceedings of the 7th Python in Science Conference*, pages 11 – 15, Pasadena, CA USA, 2008.

[6] Vladimir Iosifovich Levenshtein. Binary codes capable of correcting deletions, insertions and reversals. *Soviet Physics Doklady*, 10(8):707–710, 1966. Doklady Akademii Nauk SSSR, V163 No4 845-848 1965.

[7] F. Pedregosa, G. Varoquaux, A. Gramfort, V. Michel, B. Thirion, O. Grisel, M. Blondel, P. Prettenhofer, R. Weiss, V. Dubourg, J. Vanderplas, A. Passos, D. Cournapeau, M. Brucher, M. Perrot, and E. Duchesnay. Scikit-learn: Machine learning in Python. *Journal of Machine Learning Research*, 12: 2825–2830, 2011.

[8] Tim Sainburg, Leland McInnes, and Timothy Q Gentner. Parametric umap embeddings for representation and semisupervised learning. *Neural Computation*, 33(11):2881–2907, 2021.

[9] A. Hyvärinen and E. Oja. Independent component analysis: algorithms and applications. *Neural Networks*, 13(4):411–430, 2000. doi: https://doi.org/10.1016/S0893-6080(00)00026-5.

[10] Laurens van der Maaten and Geoffrey Hinton. Visualizing data using t-sne. *Journal of Machine Learning Research*, 9(86):2579–2605, 2008. URL `http://jmlr.org/papers/v9/vandermaaten08a.html`.

[11] Joshua B. Tenenbaum, Vin de Silva, and John C. Langford. A global geometric framework for nonlinear dimensionality reduction. *Science*, 290(5500):2319–2323, 2000. doi: 10.1126/science.290.5500.2319. URL `https://www.science.org/doi/abs/10.1126/science.290.5500.2319`.

[12] Chris Ding and Hanchuan Peng. Minimum redundancy feature selection from microarray gene expression data. *Journal of bioinformatics and computational biology*, 3(2):185–205, Apr 2005. ISSN 0219-7200. doi: 10.1142/s0219720005001004. URL `http://dx.doi.org/10.1142/s0219720005001004`.

[13] Hanchuan Peng, Fuhui Long, and C. Ding. Feature selection based on mutual information criteria of max-dependency, max-relevance, and min-redundancy. *IEEE Transactions on Pattern Analysis and Machine Intelligence*, 27(8):1226–1238, Aug 2005. ISSN 1939-3539. doi: 10.1109/TPAMI.2005.159.

[14] Tony F. Chan, Gene H. Golub, and Randall J. LeVeque. Updating formulae and a pairwise algorithm for computing sample variances, 1979.
